# [Retracted] miR‑4306 inhibits the malignant behaviors of colorectal cancer by regulating lncRNA FoxD2‑AS1

**DOI:** 10.3892/mmr.2024.13299

**Published:** 2024-08-05

**Authors:** Jinjun Ye, Jidong Liu, Tao Tang, Le Xin, Xing Bao, Yukuang Yan

Mol Med Rep 24: 723, 2021; DOI: 10.3892/mmr.2021.12362

Following the publication of this paper, it was drawn to the Editor's attention by a concerned reader that the colony formation assay data shown in Fig. 5F on p. 7 were strikingly similar to data appearing in different form in several other articles written by different authors at different research institutes, which had already been published prior to the submission of this article to the journal. In addition, possible anomalies were noted regarding the appearance of the western blots in the paper. Owing to the fact that the contentious data in the above article had already been published prior to its submission to *Molecular Medicine Reports*, the Editor has decided that this paper should be retracted from the Journal. The authors were asked for an explanation to account for these concerns, but the Editorial Office did not receive a reply. The Editor apologizes to the readership for any inconvenience caused.

